# Assessment of Cadmium (Cd) and Lead (Pb) Blood Concentration on the Risk of Endometrial Cancer

**DOI:** 10.3390/biology12050717

**Published:** 2023-05-14

**Authors:** Kaja Michalczyk, Patrycja Kupnicka, Grzegorz Witczak, Piotr Tousty, Mateusz Bosiacki, Mateusz Kurzawski, Dariusz Chlubek, Aneta Cymbaluk-Płoska

**Affiliations:** 1 Department of Gynecological Surgery and Gynecological Oncology of Adults and Adolescents, Pomeranian Medical University, 70-111 Szczecin, Poland; 2 Department of Biochemistry and Medical Chemistry, Pomeranian Medical University, Powstancow Wielkopolskich 72, 70-111 Szczecin, Poland; 3 Department of Obstetrics and Gynecology, Pomeranian Medical University, Powstancow Wielkopolskich 72, 70-111 Szczecin, Poland; 4 Department of Functional Diagnostics and Physical Medicine, Pomeranian Medical University, 70-111 Szczecin, Poland; 5 Department of Experimental and Clinical Pharmacology, Pomeranian Medical University, Powstancow Wielkopolskich 72, 70-111 Szczecin, Poland; 6 Department of Reconstructive Surgery and Gynecological Oncology, Pomeranian Medical University, Powstancow Wielkopolskich 72, 70-111 Szczecin, Poland

**Keywords:** cadmium, lead, heavy metals, carcinogens, endometrial cancer, uterine cancer

## Abstract

**Simple Summary:**

Cadmium and lead are heavy metals of carcinogenic potential, especially in excessive amounts. Their increased concentrations have been found to correlate with the risk of multiple cancers, including lung, kidney, gastrointestinal, breast, and gynecological cancers. In this study, we have examined the whole blood concentrations of cadmium and lead in patients diagnosed with uterine pathologies and endometrial cancer. We found significant differences in the cadmium/lead ratio among patients diagnosed with different pathologies, with the highest Cd levels in patients with endometrial cancer. Our results show that an increased blood cadmium concentration above the median to be a risk factor for endometrial cancer. Further research on greater populations, accounting for environmental and lifestyle heavy metal exposure, is required to validate our findings.

**Abstract:**

Background: Cadmium (Cd) and lead (Pb) are heavy metals with carcinogenic potential. Their increased concentration has been correlated with a risk of malignancies, including breast, lung, kidney, gastrointestinal, and gynecological cancers. Most of the studies have evaluated tissue heavy metal concentration. To the best of our knowledge, this is the first study to evaluate blood Cd and lead levels in different uterine pathologies and the risk of endometrial cancer. Methods: This study included 110 patients with a histopathological diagnosis of endometrial cancer, endometrial polyps, endometrial hyperplasia, uterine myoma, and normal endometrium. The patients included in the study were assessed in terms of their endometrial cancer risk factors and blood heavy metal levels. The analysis was conducted using inductively coupled plasma optical emission spectrometry. Results: There was a significant difference in the Cd and Cd/Pb ratio among the different groups of patients (*p* = 0.002), with higher a median Cd concentration among the endometrial cancer patients. The differences in Pb concentration were not significant (*p* = 0.717). There were also no differences in the Cd and Pb concentrations based on the patients’ menopausal status nor BMI index. The univariate logistic regression showed a blood cadmium concentration above the median to be associated with an increased risk of endometrial cancer (OR = 5.25; 95% CI 1.56, 17.72). No significant associations were observed between the Pb concentration or Cd/Pb ratio and endometrial cancer risk. Conclusion: The concentration of Cd varies in patients diagnosed with different uterine pathologies. Increased blood cadmium concentration seems to be a risk factor for endometrial studies. Further research on greater populations, accounting for environmental and lifestyle heavy metal exposure, is required to validate our findings.

## 1. Introduction

Endometrial cancer (EC) is the most common gynecological cancer and its incidence is constantly rising [1]. Fortunately, the majority of cases are diagnosed at an early stage and have a good patient prognosis. One of the first symptoms of endometrial cancer is post-menopausal uterine bleeding, present in more than 80% of patients, yet only 10% of post-menopausal bleeding is related to endometrial cancer [2]. Multiple patient characteristics have been associated with endometrial cancer risk, including obesity, diabetes, early menarche, late menopause, and unopposed estrogens [3]. Heavy metals are natural ingredients, which in small doses are present in all aspects of the environment, including air, water, soil, and plants. The effect of heavy metals on cancer risk has been widely studied in recent decades; however, there is still limited data on their role in gynecological malignancies and pathologies. The International Agency for Cancer Research (IARC) classified cadmium as a group 1A carcinogen, while Pb is in the second group of carcinogens (2A), with a probable cancerogenic effect on humans [4]. Their role has especially been evaluated for lung cancer prevalence [5,6,7,8]. The literature describes associations between cadmium exposure and an elevated risk of kidney [9], gastric [10], breast [11], and prostate cancer [12]. The cadmium-induced mechanism of carcinogenicity includes interference with proteins involved in the cellular response to DNA damage, dysregulation of cell growth, apoptosis resistance, and the enhanced generation of free radicals [13]. Pb was found to participate in ER signaling, activating the ERα receptor in studies evaluating the role of Pb in breast cancer [14].

The presence of heavy metals in the environment can be dangerous to human health when present in excessive amounts. The amount of heavy metals in the environment, their dietary intake, nutritional status and metabolism can be reflected by their detection in blood, tissue, skin or nail analysis [15]. Urinary and blood cadmium concentrations are found to be much lower in non-occupationally exposed people, and the sources of Cd can include exposure from air, drinking water, and food, especially in polluted areas. Occupations with the highest potential for cadmium exposure include cadmium production and refining, manufacture of Ni-Cd batteries, and cadmium pigment production. Based on research data, the geometric mean daily cadmium intake in food for the US population was assessed to be equal to 18.9 μg/day [16]. It has a long half-life, between 6 and 38 years in the kidneys and 4–19 years in the liver, and a slow elimination rate from body tissues [17]. Blood cadmium concentration is used as an indicator of both recent and cumulative exposures, while urinary cadmium have been shown to predominantly reflect cumulative exposure and the concentration of kidney cadmium [16]. In adults, the Cd level usually equals less than 0.5 μg/100 mL of blood [18]. Cadmium carcinogenicity has been reported, based predominantly on the studies demonstrating excess mortality from lung cancer among workers of cadmium recovery plats, demonstrating a dose–response relationship between the estimated cumulative exposure to cadmium and the risk of lung cancer [19]. Even though Cd does not participate in redox reactions, it has been demonstrated to induce oxidative stress both in vivo and in vitro [18]. The mechanism may be related to its inhibitory effect on antioxidant enzymes [20,21] and an inhibition of DNA-repair mechanisms, including nucleotide excision, mismatch repair, and the elimination of pre-mutagenic DNA precursors [22].

The IARC has evaluated inorganic lead and lead compounds as probably carcinogenic to humans (Group 2A). The evaluation was performed based on large cohort studies of patients exposed to inorganic lead, which revealed increased concentrations of Pb in four out of five cohorts of patients with stomach cancer [23]. Increased Pb was found to be associated with an increased risk of brain [24], gastrointestinal [25], kidney, breast [26], and prostate cancer [27]. Still, the literature provides conflicting results among the different cancer types and populations of patients as some of the studies revealed either no correlation or a negative association with cancer risk [27,28]. A study by Lim et al. [29] showed no significant association between serum Pb concentration and prostate cancer risk. A study by Menke et al. [30] revealed an association between blood Pb levels and elevated all-cause and cardiovascular death rate, but not cancer mortality. Increased cancer mortality among female workers was observed in a cohort study of lead-exposed South Korean workers. What is worth noticing is that the increased mortality was observed only in the female population and did not affect male workers [31]. The lead-related carcinogenicity mechanisms include oxidative stress and may be caused by interaction of lead with zinc finger proteins, induction of apoptosis, and an alteration of cell signaling pathways [32,33,34,35]. The sources of Pb exposure include food, water, gasoline, contaminated dust, or direct Pb exposure. Despite numerous actions to decrease global exposure to harmful substances, including Pb and Cd, there is still some relevant hazard to human health [36,37,38,39].

Some attention should be drawn to the influence of heavy metals on gynecological pathologies and malignancies due to the ability of some elements, such as Al, Cd, Ni, and Pb to act as metalloestrogens—they were discovered to have the ability to bind estrogen receptors and, through mimicking the function of estrogen receptors, they increase agonist estrogen responses [40,41]. Our study aimed to determine the concentrations of whole blood levels of Cd and Pb, assess the differences between their levels based on the patients’ characteristics and histopathological diagnosis, and determine their role as endometrial cancer risk factors.

Even though abnormal uterine bleeding is the most common symptom of endometrial cancer (present in as many as 90% of patients), the symptom lacks specificity [42]. Endometrial polyps, myomas, or endometrial hyperplasia may also be associated with uterine bleeding. Patients in advanced forms of EC may also present with nonspecific symptoms, such as abdominal distension and pain—often suggesting different possible diagnoses. This is why there is a need to develop diagnostic tools that will allow the selection of high-risk groups of patients and early EC diagnosis to allow early diagnosis and treatment. As obesity and diabetes are the main risk factors for endometrial cancer [43], dietary and nutritional factors should also be evaluated to determine their role and influence on endometrial cancer.

## 2. Materials and Methods

### 2.1. Study Subjects

This single-centre study included patients treated at the Department of Gynecological Surgery and Gynecological Oncology of Adults and Adolescents, Pomeranian Medical University. Patients with a history of uterine bleeding admitted for hysteroscopy and/or endometrial abrasion were included in the study. The study group also consisted of patients admitted for myoma removal or with a confirmed diagnosis of endometrial cancer. All patients received a histopathological diagnosis. The final study population included 110 patients: 25 patients with uterine myomas, 16 with normal endometrium, 48 with endometrial polyps, and 21 endometrial cancer patients. The study exclusion criteria were the recurrence of endometrial cancer, a previous diagnosis of any form of cancer and its treatment, and the presence of untreated or improperly managed chronic diseases. The research was conducted in accordance with the Helsinki Declaration and with the consent of the Ethics Committee of Pomeranian Medical University in Szczecin under the number KB-0012/27/2020 on 9 March 2020. All patients gave their informed consent to participate in the study and were informed about the possibility to withdraw at any time during.

### 2.2. Methodology

A 5 mL venous blood sample was collected for heavy metal analysis and placed into 2 EDTA probes (S-MonovetteEDTA K3E/2.7 mL, Sarstedt, Germany). The sample was gathered from each patient before the surgical procedure (hysteroscopy/endometrial abrasion/laparoscopy/laparotomy), based on the patients’ medical condition. Collected blood samples were stored at −80 °C until analysis. Trace metal concentration was determined using inductively coupled plasma optical emission spectrometry (ICP-OES, ICAP 7400 Duo, Thermo Scientific, Waltham, MA, USA) equipped with a concentric nebulizer and cyclonic spray chamber. Patients’ whole blood samples underwent a microwave decomposition procedure using a microwave digestion system. After proper sample defrost, and preparation, 65% HNO_3_ and H_2_O_2_ were added to the samples and the specimens were transferred into Teflon vessels and placed in the microwave digestion system (MARS5, CEM, USA). The sample digestion process was divided into two stages: an initial phase lasting 15 min (the samples were gradually heated up to 180 °C), and the second phase of 20 min, during which the temperature was maintained at 180 °C. The protocol assumed a further 5-fold dilution of the digested samples. In total, 500 μL of yttrium was added with the final standard sample concentration at 0.5 mg/L and 1 mL of 1% Triton (Triton X-100, Sigma-Aldrich, Poland). Next, sample dilution with 0.075% HNO_3_ (Suprapur, Merck, Poland) was conducted up to the volume of 10 mL. All samples were stored in the fridge at 4–8 °C until final analysis. The calibration curve was constructed using multielement standard solutions (ICP multielement standard solution IV, IX, and XVI, Merck, Kenilworth, NJ, USA). R^2^ values of each standard curve were between 0.999 and 1.000. Internal standard recovery for samples was between 92% and 102%. The accuracy of the analysis was controlled using the determination of the Cd and Pb in Bovine Muscle NIST-SRM 8414—a certified material with a known concentration (Table 1).

### 2.3. Statistical Analysis

The study participants were divided into four groups based on their histopathological diagnosis (uterine fibroma, normal endometrium, endometrial polyp, and endometrial cancer). The groups were compared using the Mann–Whitney U test, Kruskal–Wallis test, or an analysis of variance (ANOVA). The normal distribution of the data was tested with ANOVA. The patients were differentiated in terms of cadmium, lead concentration, and the patients’ characteristics (patients’ age, body weight, BMI index, menopausal status, diabetes, cigarette use). Outlier values were removed from the statistical analysis. Univariate regression analysis was performed to assess the odds ratios (OR) of endometrial cancer. A *p*-value < 0.05 was established as the statistical significance threshold. Statistical analysis was performed using R Statistical Analysis Software, R Foundation for Statistical Computing, Vienna, Austria.

## 3. Results

### 3.1. Distribution of Heavy Metal Concentration between Different Groups of Patients

**Figure 1:**
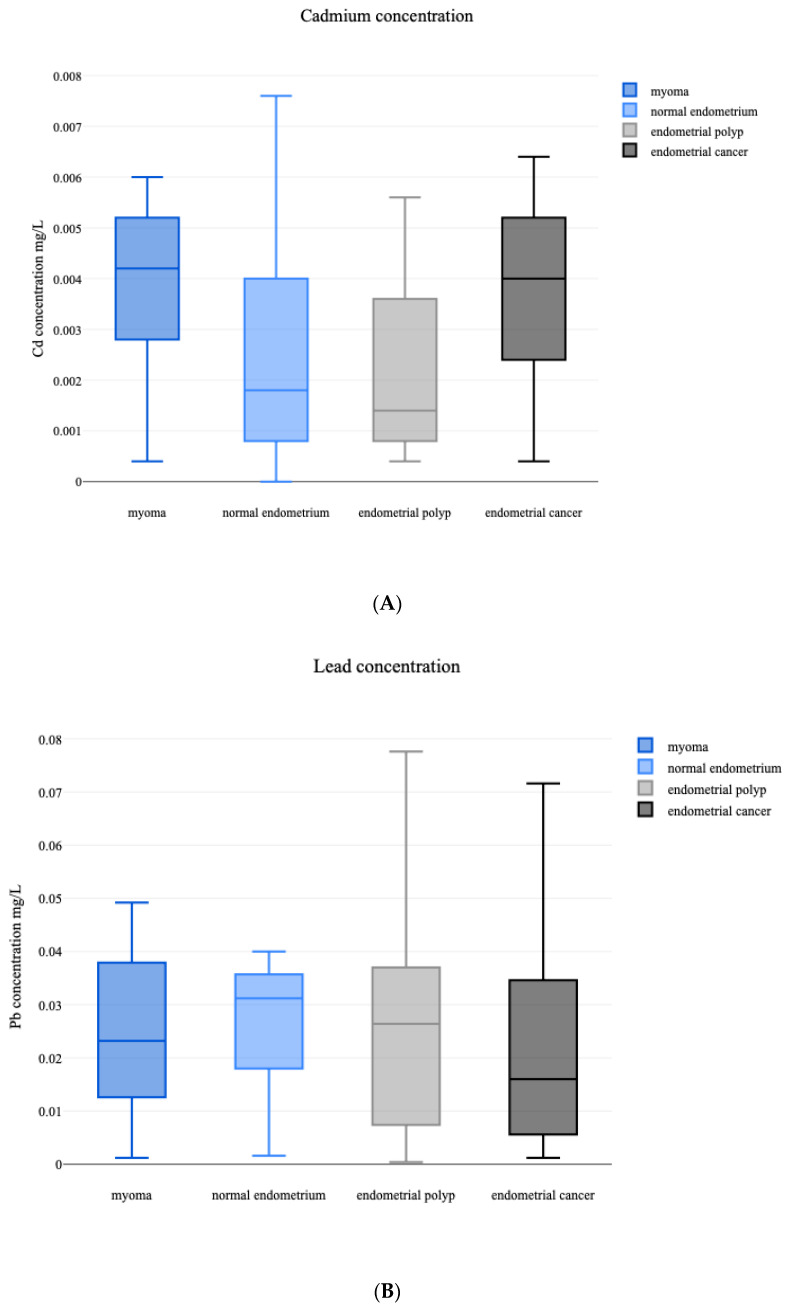

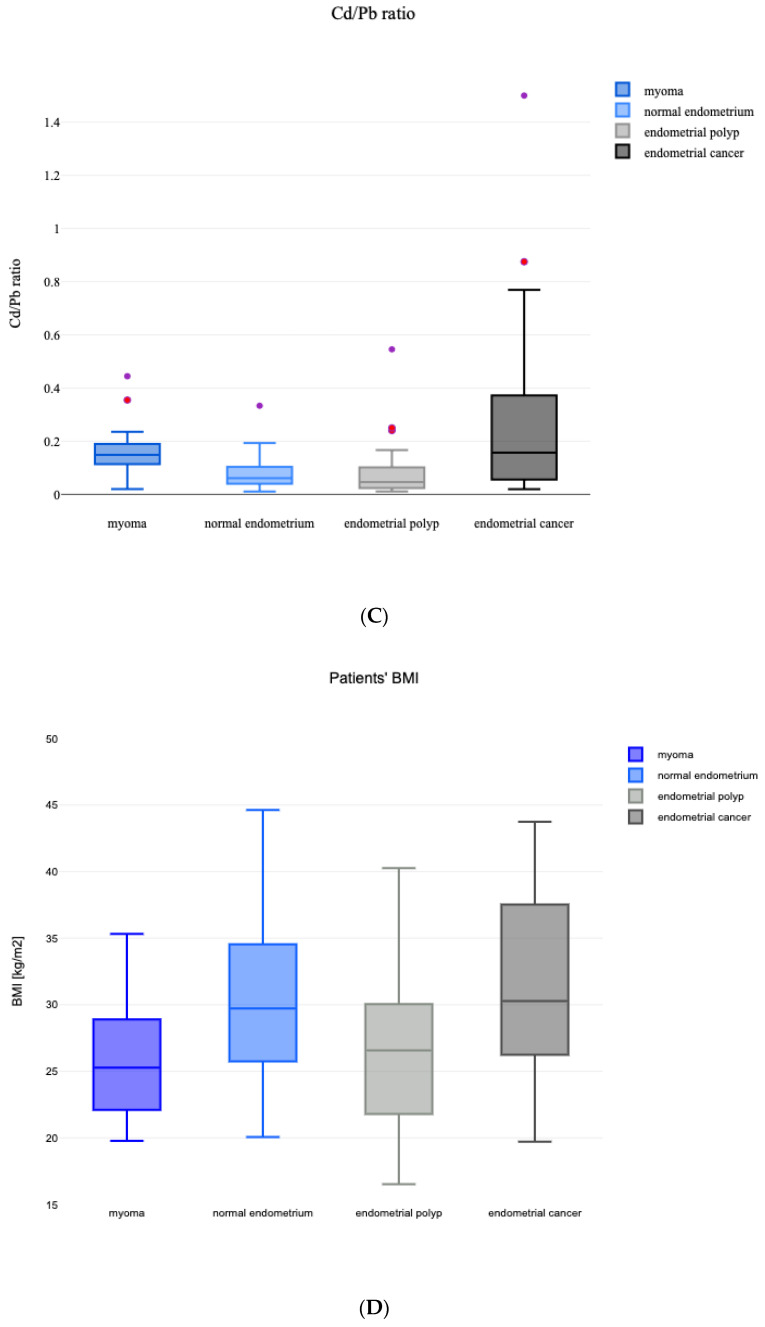

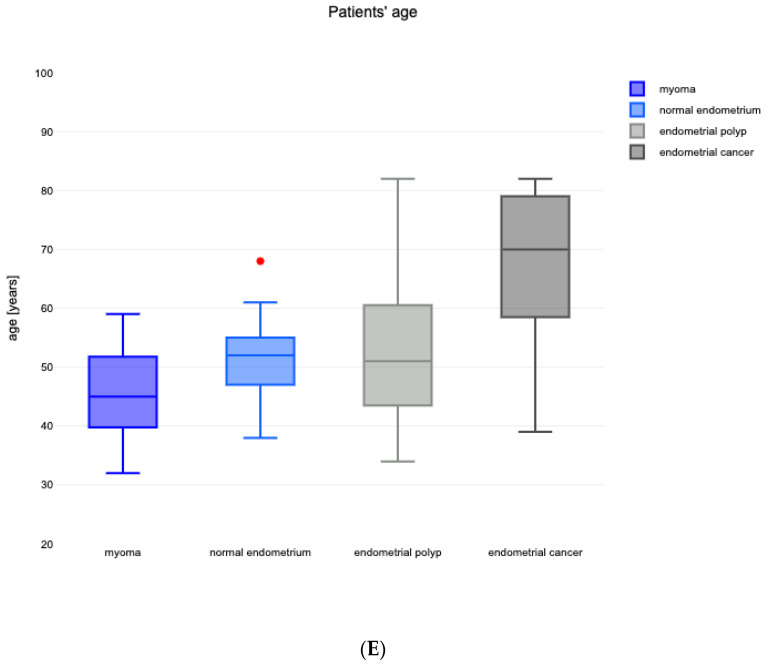
Distribution of the assessed variables between the groups. (**A**) Distribution of Cd concentration. Median Cd concentration in all of the assessed groups was 0.0028 mg/L. There are significant differences in Cd concentration between the patients diagnosed with myomas and endometrial polyps (*p* < 0.001), and endometrial polyp-endometrial cancer (*p* = 0.006). Specific results are listed in Table A1, Appendix A. (**B**) Distribution of Pb concentration. The median blood concentrations of Pb are 0.0232 mg/L in patients with myomas, 0.0312 mg/L in patients with normal endometrium, 0.0264 mg/L in endometrial polyps and 0.0160 mg/L in endometrial cancer. The differences between the assessed groups are statistically insignificant (*p* = 0.7175). (**C**) Cd/Pb ratio distribution. There are significant differences in the Cd/Pb ratio between the myoma-normal endometrium groups (*p* = 0.0294), myoma-endometrial polyp (*p* = 0.0057), normal endometrium-endometrial cancer (*p* = 0.0042), and endometrial polyp-endometrial cancer (*p* < 0.001). Specific results are listed in Table A1, Appendix A. (**D**) Patients’ BMI. In all of the assessed groups, the median BMI is 27.06. There are significant differences between the BMI of patients diagnosed with myoma-endometrial cancer (*p* = 0.0020), and endometrial polyp-endometrial cancer (*p* = 0.0187). Specific results are listed in Appendix A, Table A2. (**E**) Patients’ age. The median patients age in the whole study population is 52 years. Patients diagnosed with endometrial cancer have the highest median age of 70 years. There are significant differences between the groups myoma-endometrial polyp (*p* = 0.033), myoma-endometrial cancer (*p* < 0.001), normal endometrium-endometrial cancer (*p* = 0.003), and endometrial polyp-endometrial cancer (*p* < 0.001). Specific results are listed in Appendix A, Table A2.

Having analyzed the whole population, the median Cd level was 0.0028 mg/L, and the median Pb concentration equaled 0.2440 mg/L. An analysis of the Cd/Pb ratio was conducted. To evaluate the differences in heavy metal concentration, we compared the values between the different groups of patients based on their histopathological diagnosis (Table 2 and Figure 1).

The concentration of whole blood cadmium is significantly different across the subgroups (*p* = 0.002), with a mean rank score of 66.1 for myomas, 50.3 for normal endometrium, 40.19 for endometrial polyps, and 62.33 for endometrial cancer. The mean ranks of the following pairs are significantly different: myoma-endometrial polyp (*p* < 0.001) and endometrial polyp-endometrial cancer (*p* = 0.006). The specific results are demonstrated in Table A1, Appendix A. An analysis of the Pb concentration was conducted and showed no significant differences between the assessed groups of patients (*p* = 0.7175). Statistical analysis revealed significant differences in the Cd/Pb ratio between the different groups (*p* = 0.001). The detailed results are presented in Table A1, Appendix A.

The patients’ age and BMI were found to vary significantly between the groups. Both the median age and BMI index were the highest among the endometrial cancer patients. The lowest median age of patients was noted for the group of women diagnosed with uterine myomas (45 years). The detailed results are presented in Table A2, Appendix A.

### 3.2. Distribution of Cd and Pb Based on the Patients’ Characteristics

An analysis of the influence of patient characteristics and the presence of any comorbidities such as obesity or diabetes was conducted. We found no effect of the patients’ weight or DM type 2 on trace metal concentration. The blood Cd and Pb levels were also not affected by the patients’ menopausal status (*p* = 0.1513 and *p* = 0.5682, respectively). We have found a statistically significant influence of cigarette use on heavy metal concentration. There is a significant difference between the Pb concentrations in the smoking and non-smoking populations of patients (*p* = 0.0160). The magnitude of the difference between the value from non-smoking and the value of smoking population is small (0.25), with the observed common effect size equal to 0.86. The study results also demonstrate a significant difference in the Cd/Pb ratio between the smoking and non-smoking patients, with a medium standardized effect size of 0.37. The detailed results are presented in Table 3.

We have further analyzed the influence of the patients’ BMI on blood Cd and Pb concentration. We have differentiated the patients into four categories: underweight, normal weight, overweight and obese, based on their BMI index. The Kruskal–Wallis test indicated that the differences between the specific subgroups of patients were not big enough to be statistically significant. The detailed results are listed in Table A3 (Appendix B).

### 3.3. Endometrial Cancer Risk

For the univariate logistic regression calculation, we decided to use patients with a histopathological confirmation of normal endometrial tissue and endometrial polyps as a control group. Blood cadmium concentration above the median was associated with increased odds of an endometrial cancer diagnosis (OR = 5.25; 95% CI 1.56–17.72). In addition, an increase in the patients’ age (OR = 8.28; 95% CI 2.20–31.16) and post-menopausal status (OR = 14.77; 95% CI 1.86–117.17) were found to increase the odds of endometrial cancer. No significant associations were observed between Pb concentration or the Cd/Pb ratio and endometrial cancer risk in the studied population of patients. The specific results are presented in Table 4.

## 4. Discussion

Cadmium and lead are oxidative stress-inducing agents. Excessive amounts of any of them were demonstrated to cause DNA mutations or damage, genome instability, and cell cycle alterations, causing increased cell proliferation, leading to cancer initiation and progression [44]. The literature shows a strong correlation between the concentrations of Cd and Pb, regardless of the analyzed specimen [2,45]. Both of the particles were also found to act as estrogen-mimicking particles. As endometrial cancer is hormone-dependent, altered Cd and Pb concentrations may be associated with an increased risk of endometrial cancer. Environmental exposure and dietary habits may cause alterations in trace metal concentration, influence estrogen concentration, and/or induce carcinogenic effects that may potentially influence the risk of endometrial cancer.

Human lead absorption can be impacted by the route of exposure and was found to be inversely proportional to the exposure particle size (i.e., greater Pb exposure through lead dust inspiration via the respiratory route than via the digestive tract). The blood carries only a limited fraction of total body lead. However, it acts as the initial receptacle of the absorbed lead and causes its distribution throughout the body via blood circulation [46]. Approximately 99% of blood Pb is associated with RBCs (red blood cells), with the remaining 1% present in blood plasma [47]. The blood lead level is the most widely used measure of its exposure [46], which is why we decided to measure whole blood Pb in our study. A study by Schultze et al. investigated the absolute concentrations of heavy metals and trace elements in both serum and whole blood specimens [48]. The authors found higher absolute concentrations in whole blood compared to the patients’ serum for all of the assessed trace elements (aluminum, cobalt, chromium, mercury, manganese, nickel, lead, and zinc), apart from copper and molybdenum. They also found that the kidney function parameters (glomerular filtration rate) influence the distribution of trace metals between the patients’ RBCs and serum compartments. In our study, we only assessed the whole blood trace metal concentration, and we did not account either for the patients’ hemoglobin nor GFR. There are also different methods that can be used to determine trace metal concentration, including X-ray fluorescence spectroscopy, atomic absorption spectroscopy, inductively coupled plasma mass spectrometry and inductively coupled plasma atomic emission spectroscopy [49]. As different assays have different sensitivity and specificity, the results may vary based on the method used.

For the analysis of cadmium levels, urinary cadmium seems to be the best indicator of long-term cadmium exposure [50]. Primary cadmium accumulation occurs in the kidneys and only 0.01–0.02% of Cd is excreted daily from the human body [51]. 

A whole blood sample is a good laboratory measurement of cadmium that reflects recent cadmium exposure within the last 50 days [52,53]. As cadmium binds to the RBCs in the bloodstream, serum measurements are of limited use [54].

In this exploratory analysis, we found significant differences in the cadmium distribution between patients diagnosed with endometrial pathologies, including endometrial cancer. A similar trend was observed for the Cd/Pb ratio (*p* = 0.001), while the differences between the groups for lead (Pb) concentration were not big enough to be statistically significant. In our analysis, we found no influence of the patients’ BMI, menopausal status, or the presence of diabetes on heavy metal concentration. We have found differences in the Pb and Cd/Pb concentration between patients who did and did not declare tobacco use; however, as the population was very limited (only seven patients in the whole population sample declared smoking), the results require further analysis on a greater population sample.

The primary source of cadmium intake is cigarette smoke (both in the active and passive form), shellfish, and green leafy vegetables, with the level of Cd in food directly proportional to soil and water contamination. In previous studies, cadmium has been demonstrated to act as a metalloestrogen, mimic estrogen activity, i.e., in breast cancer cells, and bind and inhibit ERα [40,55,56]. In the univariate analysis, we found increased cadmium concentration to be a risk factor for endometrial cancer. In our study, we have confirmed the patient’s age and post-menopausal status as endometrial cancer risk factors. As increased, unopposed estrogen concentration is a risk factor for endometrial cancer [3], higher cadmium levels may furtherly increase estrogen levels and stimulate endometrial proliferation. Due to the high vascular supply to the uterus, and its endometrial lining, endometrial tissue may be sensitive to substances transported within the blood, leading to their accumulation in specific tissues and increasing the risk of carcinogenesis [57]. In our study, patients with uterine myomas also tended to have higher median Cd values. As the growth of myomas is caused by the imbalance between the cells’ proliferation and their death, favoring proliferation, it may also explain the greater accumulation of heavy metals in such patients. Endometrial biopsy is a simple, minimally invasive diagnostic procedure used to evaluate patients presenting with uterine bleedings, endometrial cancer diagnostics, and patient stratification [58]. It allows direct endometrial sampling in office conditions, without any need for patient sedation or anesthesia. The material used during the biopsy could also serve for future studies evaluating the concentration of heavy metals in patients with different endometrial pathologies. The simultaneous analysis of tissue and blood element concentrations would allow a deepening understanding of the possible correlations between trace metals and endometrial pathologies.

In the literature, we did not find any studies evaluating the influence of whole blood Cd and Pb on endometrial cancer risk nor their distribution between different uterine pathologies. There was one study evaluating blood Cd concentrations in endometrial cancer patients; however, this was evaluating the changes in Cd concentration during the duration of chemotherapy treatment, and no measurements were taken at the time of the endometrial cancer diagnosis [59]. We have found numerous researches evaluating cancer tissue heavy metal concentration, which demonstrated the accumulation of heavy metals in malignant tissues, including lung, breast, and endometrial cancer [45,60,61]. A different population-based case-control study found a significantly increased endometrial cancer risk among patients with higher urine cadmium [62]. As the concentration of Cd and Pb is greatly influenced by environmental and lifestyle factors, future studies should account for the patients’ demographics, diet, area of residence, and any environmental or occupational exposure to heavy metals. So far, the literature reports conflicting results on the association between endometrial cancer and cadmium intake [63,64,65]. A limitation of our study is that we did not perform food frequency questionnaires to determine the possible dietary intake of Cd or Pb. A study by Akesson et al. [63] suggested that cadmium intake is associated with an increased risk of endometrial cancer. The only heavy metal exposure factor we considered was the patient’s smoking status; however, very few patients declared cigarette use.

This study was conducted on a Polish population of patients of white ethnicity. As different concentrations of elements may be caused by environmental exposure, geographical location, pollution, differences in body composition, genetics, nutritional status and dietary habits, there may be differences in heavy metal concentrations between different populations of patients [66]. Due to the limited number of studies assessing heavy metals in endometrial cancer patients, we cannot compare our findings with different populations of patients. Studies on breast cancer revealed significant changes in trace metal concentrations between Korean, Canadian, and Chinese populations of patients [67].

In our study, we only conducted a singleton measurement of blood Cd and Pb concentration. A series of measurements during different timings of the menstrual cycle and/or different phases of diagnosis and treatment would provide further insight into the study. It would also be interesting to determine changes in Cd concentration after the surgical treatment of EC patients. The effect of other trace elements could also be studied to determine if there are any other correlations between any trace metals and endometrial cancer or endometrial pathologies.

## 5. Conclusions

In conclusion, our data suggest increased blood cadmium concentration as a risk factor for endometrial cancer. Further studies on greater population samples are needed to evaluate the role of heavy metals in different endometrial pathologies. If the study results are confirmed, Cd may serve as a factor to eliminate, lowering the potential odds of cancer risk.

## Figures and Tables

**Table 1:** Results of the analysis of the reference material NIST-SRM 8414. The results are presented as the mean ± SD.

Element	Reference Values (mg/kg)	Obtained Values, *n* = 3 (mg/kg)	% of Reference Value
Cd	0.013 ± 0.01	0.018 ± 0.004	138%
Pb	0.38 ± 0.24	0.42 ± 0.11	111%

**Table 2:** Comparison of the selected variables between the study groups.

Variable	Study Population (N = 110)	Myoma (N = 25)	Normal Endometrium (N = 16)	Endometrial Polyp (N = 48)	Endometrial Cancer (N = 21)	*p*-Value
Cd (mg/L)	0.0028 (0.0012; 0.0044)	0.0042	0.0018	0.0014	0.00	0.0020
Pb (mg/L)	0.244 (0.0088; 0.0373)	0.0232	0.0312	0.0264	0.0160	0.7175
Cd/Pb ratio	0.0925 (0.0286; 0.1811)	0.1456	0.0439	0.0400	0.1569	0.0011
Age (years)	52 (45; 62)	45	52	51	70	<0.0001
Weight (kg)	72.0 (60.0; 86.0)	69.8 ± 13.4	79.1 ± 15.0	71.1 ± 15.5	78.7 ± 21.2	0.1284 *
BMI (kg/m^2^)	27.06 (22.76; 32.26)	25.28	29.72	26.56	30.27	0.0252

Data are reported as the mean ± standard deviation or median. The groups were compared using ANOVA followed by Tukey’s multiple test * or the Kruskal–Wallis test with Dunns’ post hoc multiple comparison test. IQR (interquartile range) is listed in brackets. N = number of patients. *p* values < 0.05 are considered significant.

**Table 3:** Comparison of median trace metal concentration based on the patients’ characteristics and comorbidities.

	Cd (mg/L)	Pb (mg/L)	Cd/Pb Ratio
Before menopause [*n* = 45]	0.0036 ± 0.0018	0.0300 ± 0.0224	0.1200 ± 0.2875
After menopause [*n* = 63]	0.0024 ± 0.0021	0.0228 ± 0.0171	0.1034 ± 0.2015
*p*-value	0.1513	0.5682	0.8494
BMI < 25 kg/m^2^ [*n* = 35]	0.0024 ± 0.0021	0.0348 ± 0.0164	0.1078 ± 0.0886
BMI ≥ 25 kg/m^2^ [*n* = 69]	0.0028 ± 0.0020	0.0188 ± 0.0187	0.1456 ± 0.2401
*p*-value	0.6576	0.1239	0.1030
No diabetes [*n* = 93]	0.0028 ± 0.0020	0.0268 ± 0.0201	0.1034 ± 0.1782
Diabetes type 2 [*n* = 15]	0.0028 ± 0.0021	0.0204 ± 0.0163	0.1609 ± 0.1851
*p*-value	0.4981	0.3782	0.0764
Non-smoking [*n* = 101]	0.0028 ± 0.0020	0.0264 ± 0.0188	0.1034 ± 0.1318
Smoking [*n* = 7]	0.0028 ± 0.0024	0.0024 ± 0.0077	1.0000 ± 2.4080
*p*-value	0.7078	0.0160	0.0011

The values are listed as the median ± SD. N = number of patients. The groups were compared using Mann–Whitney U test. *p*-values < 0.05 were considered significant.

**Table 4:** Univariate logistic regression.

	Characteristics	OR	95%CI	*p*-Value
Cd	>Q1	1.70	0.34–8.55	0.5195
	>Q2	5.25	1.56–17.72	0.0075
	>Q3	1.92	0.63–5.80	0.2496
Pb	>Q1	0.87	0.26–2.90	0.8163
	>Q2	0.37	0.12–1.13	0.0802
	>Q3	-	-	-
Cd/Pb	>Q1	4.47	0.52–38.22	0.1716
	>Q2	3.50	0.85–14.33	0.0815
	>Q3	3.19	0.78–13.06	0.1071
Age *		8.28	2.20–31.16	0.0018
Weight *		1.27	0.46–3.55	0.6452
BMI *		2.189	0.77–6.24	0.1428
Menopause		14.77	1.86–117.17	0.0108
Smoking		2.69	0.55–13.20	0.2225
Diabetes type 2		2.944	0.99–8.78	0.5260

* Concentration > median, Q1—lower quartile, Q2—median, Q3–upper quartile; *p* values < 0.05 were considered significant.

## Data Availability

The data presented in this study are available on request from the corresponding author. The data are not publicly available due to ethical restrictions.

## Appendix A

**Table A1:** Differences in the trace metal concentrations between specific groups of patients.

Variable	Pair *	Mean Rank Difference	Z	Critical Value	*p*-Value
Cd	X1–X2	18.94	1.95	25.60	0.051
	X1–X3	25.91	3.40	20.13	<0.001
	X1–X4	3.78	0.42	23.79	0.675
	X2–X3	6.96	0.79	23.16	0.428
	X2–X4	−15.17	1.50	26.61	0.133
	X3–X4	−22.13	2.76	21.19	0.006
Cd/Pb	X1–X2	18.19	2.18	22.04	0.0294
	X1–X3	18.48	2.76	17.64	0.0057
	X1–X4	−4.04	0.54	19.66	0.5877
	X2–X3	0.28	0.04	20.52	0.971
	X2–X4	−22.24	2.63	22.28	0.0042
	X3–X4	−22.52	3.31	17.94	<0.001

* X1—myoma group, X2—normal endometrium group, X3—endometrial polyp group, X4—endometrial cancer; *p* values < 0.05 were considered significant.

**Table A2:** Differences in the patients’ age and BMI index between the specific subgroups.

Variable	Pair *	Mean Rank Difference	Z	Critical Value	*p*-Value
Age	X1–X2	−17.00	1.6674	26.9	0.095
	X1–X3	−16.6	2.1366	20.6	0.033
	X1–X4	−48.0	5.1103	24.8	<0.001
	X2–X3	0.35	0.0340	24.0	0.969
	X2–X4	−31.0	2.9543	27.7	0.003
	X3–X4	−31.4	3.8348	21.6	<0.001
BMI	X1–X2	−19.51	1.95	26.39	0.0511
	X1–X3	−2.31	0.30	20.43	0.7651
	X1–X4	−21.13	2.29	24.35	0.0220
	X2–X3	17.20	1.94	23.44	0.0529
	X2–X4	−1.617	0.16	26.92	0.8741
	X3–X4	−18.81	2.35	21.11	0.0187

* X1—myoma group, X2—normal endometrium group, X3—endometrial polyp group, X4—endometrial cancer; *p* values < 0.05 were considered significant.

## Appendix B

**Table A3:** Distribution of Cd and Pb levels among different BMI categories.

Variable	Underweight (<18.5 kg/m^2^)	Normal Weight (18.5–25 kg/m^2^)	Overweight (25–30 kg/m^2^)	Obese (≥30 kg/m^2^)	*p*-Value
Cd (mg/L)	0.0010	0.0028	0.0024	0.0030	0.4412
Pb (mg/L)	0.0408	0.0300	0.0208	0.0232	0.2176
Cd/Pb ratio	0.0278	0.1078	0.0717	0.1562	0.0776

Data are reported as the median. The groups were compared using the Kruskal–Wallis with Dunns’ post hoc multiple comparison test; *p* values < 0.05 were considered significant.
